# Subcutaneous Emphysema as a Complication of Tonsillectomy: A Systematic Literature Review and Case Report

**Published:** 2018-01

**Authors:** Panagiotis Saravakos, Margaritis Taxeidis, Ioannis Kastanioudakis, Oliver Reichel

**Affiliations:** 1 *Department of Otorhinolaryngology, Head and Neck Surgery, Siloah St. Trudpert Hospital, Pforzheim, Germany.*; 2 *Department of Otorhinolaryngology Head and Neck Surgery, University Hospital of Ioannina, Ioannina, Greece.*

**Keywords:** Tonsillectomy, Mediastinal Emphysema, Subcutaneous Emphysema

## Abstract

**Introduction:**

Subcutaneous and mediastinal emphysema is a rare complication after tonsillectomy. This case presentation and literature review summarizes the existing literature on this unusual complication.

**Materials and Methods:**

This study presents a case of a 21-year-old man who developed a cervical subcutaneous emphysema 6 days after tonsillectomy, whereby conservative treatment produced spontaneous resolution. A proper analysis of this case also required undertaking a systematic search in MEDLINE/PubMed and SCOPUS electronic databases concerning this rare complication, without language restrictions.

**Results:**

Based on our criteria, we identified 41 reports including 43 individual cases, in which patients were mostly young and equally distributed between the genders (18 males and 23 females, two unknown). The treatment was mainly conservative and consisted of observation and/or antibiotic therapy.

**Conclusion:**

Subcutaneous or mediastinal emphysema is an uncommon complication after tonsillectomy. It is important that clinicians become aware of this rare complication, which requires a close monitoring of the patient.

## Introduction

Tonsillectomy is one of the most common surgical procedures in otorhinolaryngology. Post-tonsillectomy, primary or secondary bleeding remains the most significant risk, but other common complications include damage to teeth, otalgia, odynophagia, throat infection, nausea, and vomiting (1,2). Less common immediate or intermediate complications following tonsillectomy or adenotonsillectomy include jugular vein thrombosis, Grisel syndrome (nasopharyngeal torticollis), mandible condyle fracture, infection, nasopharyngeal stenosis and velopharyngeal insufficiency (3,4). Moreover, another rare but potentially life-threatening complication following tonsillectomy is a cervicofacial subcutaneous emphysema (5-7). 

In our case presentation, we attempted to summarize the incidence, clinical characteristics and proper therapeutic considerations of the post-tonsillectomy subcutaneous and/or mediastinal emphysema and to alert the medical community considering this rare complication. We also performed a systematic review of the literature aiming to collect case reports, summarize knowledge, and provide a basis for future studies.

## Materials and Methods

*Case presentation*

A 21-year-old man suffering from recurrent chronic tonsillitis was admitted to our department for elective tonsillectomy. The medical, surgical and family history of the patient were unremarkable, and the preoperative physical examination revealed no abnormalities. Tonsillectomy was performed under general anesthesia with orotracheal intubation, using microscope-assisted extracapsular removal of the tonsils with the tonsil dissector. Hemostasis was achieved using cautery with bipolar forceps. The report described moderate tonsillar adhesions and no remarkable intraoperative bleeding.

**Fig 1:**
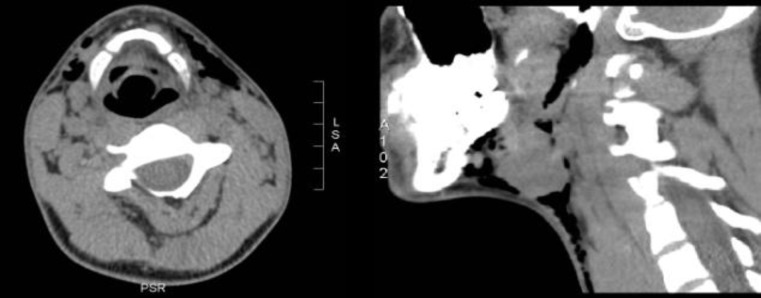
Computed tomography scan in the axial and sagittal planes showing air in the cervical subcutaneous tissues on both sides of the neck, especially on the left. There is also interruption of the air column in the left tonsillar lodge

On the third postoperative day, the patient complained of swelling in the left submandibular area. He denied symptoms of coughing or dyspnea. A physical examination revealed a painless left cervical swelling with crepitus extending from the left submandibular to the left supraclavicular region. There were no signs of local infection, and oral examination revealed a normal wound healing process without any obvious mucosal tears at the tonsillar fossa. A computer tomography (CT) scan demonstrated a subcutaneous emphysema on both sides of the neck, not obstructing the airway (Fig.1).

We administered broad-spectrum antibiotics with cephalosporin (cefuroxime) and metronidazole, as well as cough suppressants and laxatives. We instructed the patient to refrain from coughing and activities that would increase intrathoracic pressure (e.g. the Valsalva maneuver). The further course was uneventful, and the subcutaneous emphysema progressively resolved over the following days. The patient was discharged 10 days after surgery, whereby follow-up examinations revealed no further abnormal findings.

*Literature review methods*

We researched appropriate literature using a systematic computerized search in MEDLINE (via PubMed) and SCOPUS electronic databases. The keywords used included “tonsillectomy”, “adenotonsillectomy” and “emphysema” in various combinations, but with no language restrictions. We reviewed the references in each article and manually crosschecked them to ensure we included all applicable literature. Our study included all case reports containing original data relating to subcutaneous and/or mediastinal emphysema after performing tonsillectomy or combined adenotonsillectomy. However, we excluded pneumoperitoneum cases without mediastinal or cervical emphysema, and excluded subcutaneous emphysema cases after dental procedures. Descriptive statistical analysis was performed to describe and summarize data. A t-test for independent samples was performed to analyze differences between the two gender groups (mean values and standard deviation, SD). A P-value<0.05 was considered statistically significant. All statistical procedures were conducted using IBM SPSS Version 20.0. 

## Results

**Fig 2:**
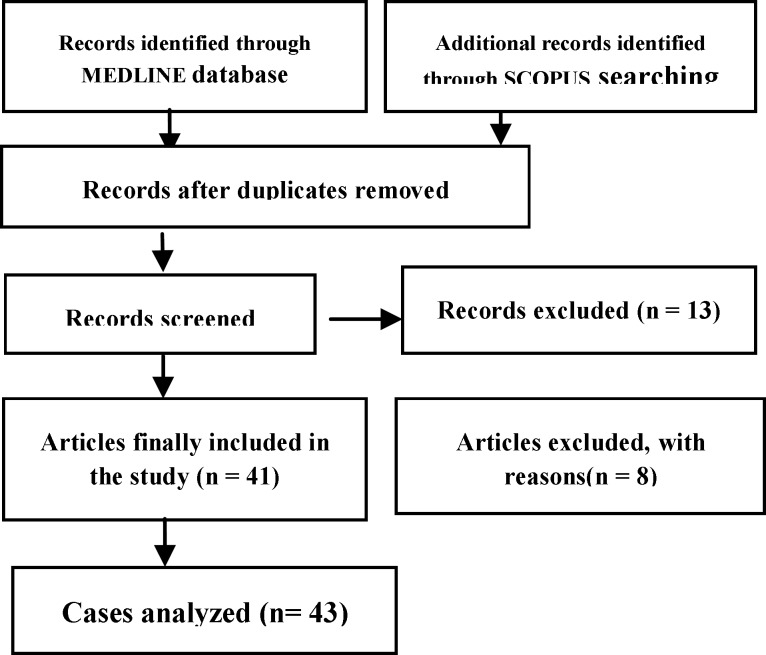
Flow chart presenting the design of the systematic review.

**Fig 3:**
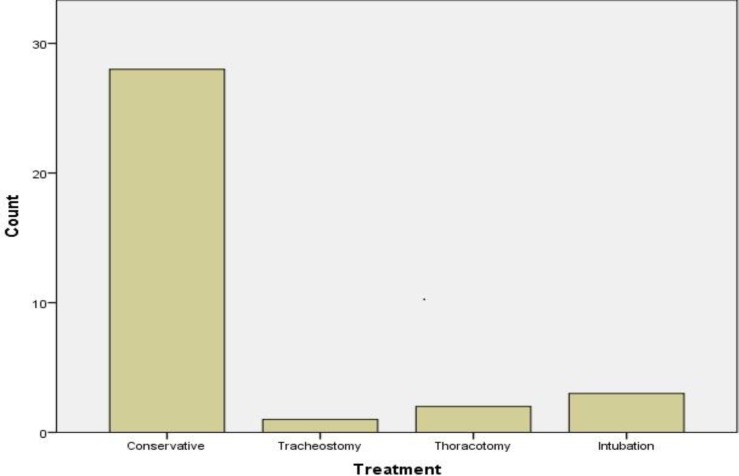
Box plot showing the median and range of the age concerning the occurrence of the subcutaneous emphysema. A statistical analysis showed no significant difference for age of occurrence between the genders, with a p-value of 0.525

As depicted in the flow chart (Fig.2), we found 66 records in the medical literature, eliminating 13 after evaluating the title and the abstract as insufficient to meet the inclusion criteria. We also excluded nine other records as they did not provide sufficient information concerning patient characteristics and therapeutic modality. Ultimately, we maintained the remaining 41 records (43 individual cases) for this study, as chronologically presented in Tables 1 and 2 (5, 6, 8-44). The first report was published in 1923, and we analyzed all subsequent reports. Patient ages ranged widely from 2 to 55 years (with a mean of 24.5, median 22, standard deviation 15.9) and included 18 males and 23 females (2 missing data) (Fig. 3). 

**Fig 4:**
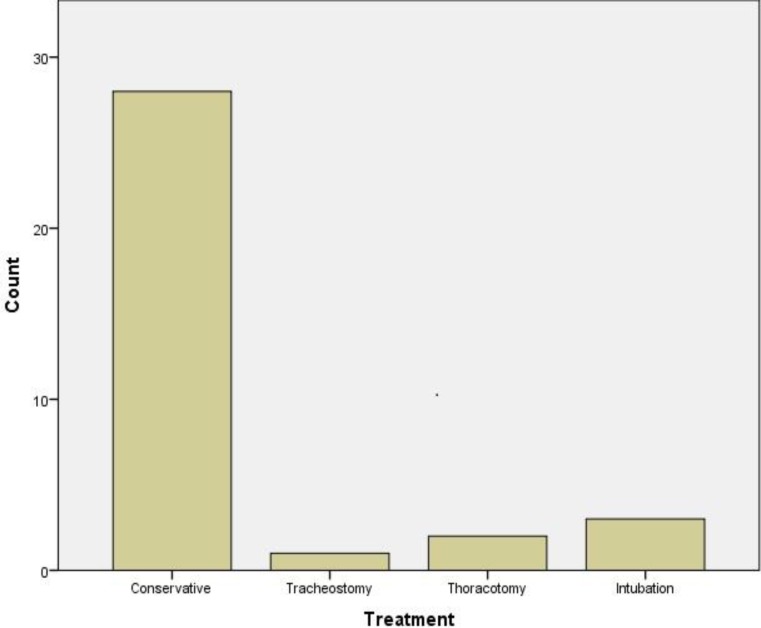
Histogram presenting the different methods of treatment of the subcutaneous emphysema. The treatment was either conservative, including observation and/or antibiotic therapy or interventional/surgical with re-intubation, tracheostomy or thoracotomy. Two patients required a thoracotomy, three patients a re-intubation and one patient required a temporary tracheostomy

**Fig 5:**
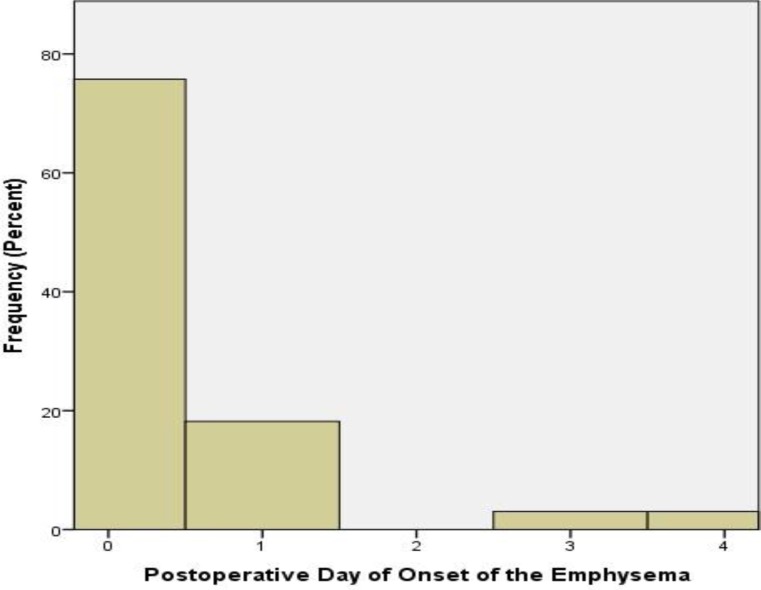
Time of occurrence of subcutaneous emphysema after tonsillectomy. In the majority of cases, the complication occurred on the operation day or on the first postoperative day

The treatment was either conservative including observation and/or antibiotic therapy or interventional/surgical with re-intubation, tracheostomy or thoracotomy. More specifically, two cases required a thoracotomy, three cases required a re-intubation, and in one case a temporary tracheostomy was needed (Fig.4). Concerning the time of the onset of emphysema, in the majority of cases (75%) this occurred on the day of surgery, while only 5% of cases developed emphysema after the third postoperative day (Fig.5).

**Table 1:** Reported cases of subcutaneous or mediastinal emphysema as a complication of tonsillectomy (years 2000–2015

**Patient #**	**Year of publication**	**Author**	**Patient age**	**Gender**	**Time of onset**	**Treatment**
1	2015	Tran et al. (40)	30	f	4 days	Antibiotics
2	2014	Bizaki et al. (12)	29	f	14 hours	Antibiotics
3	2014	Yelnoorkar et al. (43)	18	m	36 hours	Antibiotics
4	2014	Al Jabr et al. (8)	43	m	4 days	Antibiotics
5	2013	Sachdeva (35)	54	m	1 hour	Antibiotics
6	2013	Koukoutsis et al. (45)	21	f	First postoperative hours	Antibiotics
7	2012	Randrup et al.		f		
8	2011	Al Omari et al. (9)		f		
9	2011	Wahid et al. (41)				
10	2011	Durchholz et al. (14)				
11	2010	Kim et al. (18)	36	f	First day	Antibiotics
12	2009	Al-Layla et al. (10)	20	f	Immediately	Antibiotics
13	2009	Hung et al. (6)	37	m	Immediately	Intubation, antibiotic, parenteral nutrition
14	2009	Piotrowsi et al. (29)	6	m		Intubation, thoracotomy, antibiotics
15	2009	Melgaard (46)	45	f		?
16	2007	Siedek et al. (37).	20	f	Immediately	Antibiotics
17	2006	Villagra Siles et al. (47)	49	f	First day	Antibiotics
18	2005	Patel et al. (28)	31	m	6 hours	Antibiotics
19	2005	Lima et al. (20)	25	m	4 hours	Antibiotics
20	2005	Gillot et al. (5)	52	f	2 hours	Intubation, antibiotics
21	2005	Gillot et al. (5)	43	f	2 days	Cervical surgical drainage (abscess),intubation, antibiotics
22	2005	Shine et al. (36)	7	f	20 minutes	Antibiotics
23	2004	Stewart et al. (38)	31	f	Immediately	Tracheostomy, antibiotics
24	2004	Watanabe et al. (42)	24	m	First day	Antibiotics
25	2003	Nishino et al. (27)	55	f	8 hours	Antibiotics
26	2003	Fechner et al. (15)	21	f	Intraoperatively	Antibiotics
27	2003	Marioni et al. (23)	34	f	5 hours	Antibiotics
28	2001	Miman et al. (26)	11	m	Immediately	Antibiotics
29	2000	Tanaka et al. (48)	18	m	3 hours	Conservative

? =information not available; m=male; f=female Entries not included in the descriptive statistics due to missing data are represented in italics

**Table 2:** Reported cases of subcutaneous or mediastinal emphysema as a complication of tonsillectomy (years 1924–1999

**Patient #**	**Year of publication**	**Author**	**Patient age**	**Gender**	**Time of onset**	**Treatment**
1	1998	Ruiz Gómez et al. (34)	43	m		
2	1997	Hampton et al. (17)				
3	1997	Hampton et al. (17)	?			
4	1997	Lopez Gonzalez et al. (21)	Child			
5	1997	Braverman et al. (13)	22	f	2 hours	Conservative
6	1990	Rous et al. (33)	8			
7	1986	Giussan et al. (16)				
8	1978	Luk'ianenko et al. (22)				
9	1973	Prupas et al. (31)	22	m	14 hours	Observation
10	1962	Pratt et al. (30)	9	f		Thoracotomy
11	1960	Zivkovic et al. (44)				
12	1958	Andersen et al. (11)	8	f	1 day	Conservative
13	1957	McGreevy et al. (24)				
14	1955	Ferguson et al. (49)	4	f	3 hours	Thoracotomy, blood transfusion
15	1954	Knutson et al. (50)	6	m		Antibiotics
16	1953	Kazantseva et al. (51)				
17	1953	Silverman et al. (52)	8	m	10 hours	Antibiotics
18	1952	Tezel et al. (53)				
19	1936	Baker et al. (54)	27	f	1 day	Observation
20	1930	von Hofe (55)	2	m	Immediately	Conservative
21	1930	von Hofe (55)	4	f	Immediately	Conservative
22	1930	von Hofe (55)	2,5	m	Immediately	Conservative
23	1923	Stein (56)	7	m	Immediately	
24	1924	Rosenbaum (57)	35	f	Immediately (10 minutes)	Conservative

? =information not available; m=male; f=female Entries not included in the descriptive statistics due to missing data are represented in italics

## Discussion

Subcutaneous emphysema is a rare complication following procedures in the oropharyngeal or tracheobronchial area. The exact pathogenic mechanism remains unclear, but there are two main theories.

First, an important potential cause is the anesthetic procedure itself. More specifically, emphysema may result from a tear in the tracheobronchial mucosa or laryngeal trauma occurring during passage of the endotracheal tube, through overinflation of the cuff of the endotracheal tube, by excessively high alveolar pressure, or because of a malfunction of the ventilator during intubation. Congenital dehiscence in the mucosa, such as bullae, clefts or laryngoceles may also predispose to development of postoperative surgical emphysema (6, 58-60). Iatrogenic tracheal injury due to orotracheal intubation is a rare entity, with an incidence of approximately 0.005% when a single-lumen tube is used (61). In cases of airway lesions, the air enters the mediastinum and, from there, the subcutaneous tissues of the neck. However, many cases, including the present case report, describe an isolated subcutaneous emphysema without mediastinal involvement, which cannot be easily explained by the above-mentioned mechanism. Furthermore, one case was reported after performing tonsillectomy under local anesthesia, supporting the pathogenic theory of penetration of the tonsillar bed during tonsillar removal or during injection of local anesthetic (33). In our case, there was no difficulty in the tracheal intubation.

**Fig 6:**
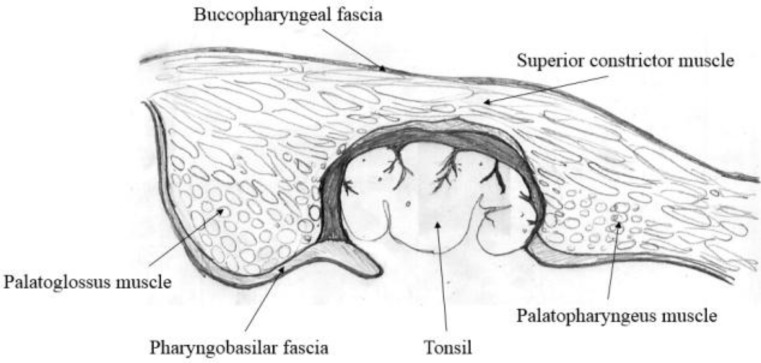
Palatine tonsil and its surrounding fasciae

The second pathogenic mechanism involves a mucosal and muscular tear of the pharynx and air entry in the parapharyngeal space. This mechanism involves the structures intervening between the tonsillar fossa and the parapharyngeal space as well as the mediastinum. The tonsillar capsule is a portion of the pharyngobasilar fascia covering the deep aspect of the tonsil and extending into it to form septa that carry the nerves and blood vessels. Loose connective tissue attaches the tonsillar capsule, covering the pharyngeal muscles, which allows easy removal by dissection of the capsule from the muscle superficial to the pharyngobasilar fascia in the peritonsillar space. However, in the case of recurrent tonsillitis, adhesions to the underlying muscle make the extracapsular dissection difficult. This fascia covers the superior constrictor muscle. Deeper lies the buccopharyngeal fascia, a part of the visceral division of the middle layer of the deep cervical fascia. The buccopharyngeal fascia separates the pharyngeal muscular wall from spaces that laterally surround it, covering the constrictor muscles and the buccinator muscle (62) (Fig.6). A rough preparation of the tonsillar capsule can damage the buccopharyngeal fascia, allowing air to enter into the parapharyngeal space. Furthermore, the visceral layer extends into the thorax around the trachea and esophagus. Consequently, the emphysema can extend into the superior mediastinum, causing a pneumomediastinum with symptoms of respiratory deficiency.

After taking into consideration the underlying pathophysiological mechanisms, the question remains as to what measures the surgeon should employ to reduce the possibility of a subcutaneous emphysema after a tonsillectomy. Preventive measures include careful surgical dissection of the tonsils to prevent damage to the parapharyngeal muscles, as well as avoiding activities that increase pharyngeal pressure, such as coughing, vomiting, sneezing, nose blowing, physical exertion, and post-extubation manual ventilation (20). The typical clinical finding is crepitation, but a CT-scan best detects extension of the emphysema. Differential diagnosis includes other causes of rapid-onset neck swelling, such as hemorrhage, hematoma and allergic reactions (17). Another important clinical condition, which should be taken into consideration as it has been infrequently described as a complication of tonsillectomy, is necrotizing fasciitis. Necrotizing fasciitis is soft-tissue infection characterized by rapidly progressing inflammation and necrosis of the subcutaneous fascial tissues. It has a particularly high mortality rate due to the proximity of many vital anatomical structures. The lack of relevant risk factors, clinical signs of erythema, fever and painful swelling, as well as laboratory and radiological findings may help distinguish these two different pathologic conditions (63).

The treatment of manifested subcutaneous emphysema is usually conservative and includes a regular cardiopulmonary assessment and administration of broad-spectrum antibiotics. We consider the administration of antibiotics obligatory to prevent contamination and infection from the oral cavity. This is because cervical emphysema may be associated with bacteria spreading in the mediastinum or the development of cervical necrotizing fasciitis (64). Supplemental oxygen may facilitate absorption of nitrogen from air accumulating in the emphysematous cavity in a favorable downward concentration gradient (8). Avoiding pressure elevation can help to reduce the occurrence of emphysema following a tonsillectomy and the patient should be advised to avoid sneezing with a closed mouth, blowing the nose and other activities that can increase intrathoracic or intraoral pressure (e.g. the Valsalva maneuver). In this rationale, medications that suppress coughing and vomiting can be prescribed. In cases of respiratory failure, measures securing the airway by means of intubation or tracheostomy are favorable. In rare cases, a mediastinal expansion of the emphysema may require a thoracotomy. When the mucosal rupture is evident, a careful suture of the damaged mucosa is also advisable.

Despite mounting information regarding this subject, medical professionals must acknowledge important limitations of the data. Case reports and case series are classified as Level IV evidence according to the National Health and Medical Research Council system for assessing level of evidence (46), and thus are rarely conclusive. It should also be noted that in many records, not all details of the cases appear (e.g. the surgical method of tonsillectomy, or the exact nature of the conservative treatment), making this systematic review of case reports more a case-series study offering material for future observational or experimental studies.

## Conclusion

Subcutaneous emphysema is a rare but potentially life-threatening complication of tonsillectomy. The medical community should be aware of this complication. A follow-up of the patient and appropriate cooperation between anesthesiologists and surgeons can help prevent this complication progressing.
